# piRNA-14633 promotes cervical cancer cell malignancy in a METTL14-dependent m6A RNA methylation manner

**DOI:** 10.1186/s12967-022-03257-2

**Published:** 2022-01-29

**Authors:** Qi Xie, Zhen Li, Xiao Luo, Dan Wang, Yao Zhou, Jingge Zhao, Suhua Gao, Yongguang Yang, Wanying Fu, Lingfei Kong, Tingyi Sun

**Affiliations:** 1 Department of Pathology, Henan Provincial People’s Hospital, People’s Hospital of Zhengzhou University; People’s Hospital of Henan University, Zhengzhou, Henan 450003 People’s Republic of China; 2 Henan International Medical Center, Henan Provincial People’s Hospital, People’s Hospital of Zhengzhou University; People’s Hospital of Henan University, Zhengzhou, Henan 450003 People’s Republic of China; 3 Department of Neurologyeurology, Henan Provincial People’s Hospital, People’s Hospital of Zhengzhou University; People’s Hospital of Henan University, Zhengzhou, Henan 450003 People’s Republic of China; 4 Department of Scientific Research and Discipline Construction, Henan Provincial People’s Hospital, People’s Hospital of Zhengzhou University; People’s Hospital of Henan University, Zhengzhou, Henan 450003 People’s Republic of China

**Keywords:** Cervical cancer, piRNA-14633, Methyltransferase-like protein 14, m6A RNA methylation, Malignancy

## Abstract

**Background:**

Cervical cancer (CC) is one of the most common gynecological tumors that threatens women's health and lives. Aberrant expression of PIWI-interacting RNA (piRNA) is closely related with a range of cancers and can serve as a tumor promoter or suppressor in proliferation, migration and invasion. In this study, the aim was not only to discover differential expression of piRNA in CC tissue (CC cells) and normal cervical tissue (normal cervical epithelium cells), but also to investigate the biological function and action mechanism of piRNA in CC.

**Methods:**

The DESeq2 approach was used to estimate fold change in piRNA between CC tissue and normal cervical tissue. The relative expressions of piRNAs (piRNA-20657, piRNA-20497, piRNA-14633 and piRNA-13350) and RNA m6A methyltransferases/demethylases were detected using RT-qPCR. After intervention with piRNA-14633 and METTL14 expression, the viability of CaSki cells and SiHa cells was detected by CCK8. CC cell proliferation was detected by colony formation assay. Apoptosis rate and cell cycle were detected by flow cytometry. Transwell assay was performed to detect cell migration and invasion. EpiQuik m6A RNA Methylation Quantification Kit was used to evaluate m6A RNA methylation levels. Expression of methyltransferase-like protein 14 (METTL14), PIWIL-proteins and CYP1B1 were detected by RT-qPCR and western blot. The effect of piRNA-14633 on METTL14 was evaluated by a dual-luciferase reporter assay. The in vivo effects of piRNA-14633 on CC was assessed by nude mice experiments.

**Results:**

piRNA-14633 showed high expression in CC tissues and cells, piRNA-14633 mimic (piRNA-14633 overexpression) promoted viability, proliferation, migration and invasion of CaSki cells and SiHa cells. Besides, piRNA-14633 mimic increased m6A RNA methylation levels and METTL14 mRNA stability. Results of dual luciferase reporter assays indicated that METTL14 was a directed target gene of piRNA-14633. Knockdown of METTL14 with siRNA attenuated proliferation, migration and invasion of CC cells. piRNA-14633 increased CYP1B1 expression, while silencing of METTL14 impaired its expression. The effect of piRNA overexpression on METTL14 expression has concentration-dependent characteristics. Results from in vivo experiment indicated that piRNA-14633 promoted cervical tumor growth.

**Conclusion:**

piRNA-14633 promotes proliferation, migration and invasion of CC cells by METTL14/CYP1B1 signaling axis, highlighting the important role of piRNA-14633 in CC.

**Supplementary Information:**

The online version contains supplementary material available at 10.1186/s12967-022-03257-2.

## Background

Cervical cancer (CC) is the third common cancer in women worldwide, with 604,127 new cases and 341,831 deaths in 2020 [1]. High-risk human papillomavirus (HPV) infection is one of the main causes of CC. Currently, early-stage CC is treated with surgery, chemotherapy or radiotherapy; treatments for late-stage CC involves chemotherapy and radiotherapy, accompanied by poor prognosis [2, 3]. Unfortunately, the higher recurrence rate is major obstacles to the long-term use of chemotherapy and radiotherapy for CC [4, 5]. It is therefore of great interest to search for novel markers of CC to provide new ideas and therapeutic targets for clinical examination, early diagnosis and prevention.

Piwi-interacting RNA (piRNA) was first identified in 2006 and is defined as a small non-coding RNAs that binds specifically to PIWI family proteins in testicular germ cells [6, 7]. PiRNA is approximately 30 nucleotides in length and regulates transposon silencing and mRNA turnover, both of which are critical for gametogenesis and germ cell functions [8–11]. Recent studies have shown that piRNA is effectively expressed in both normal somatic cells and cancer cells [12]. PiRNA has also been shown to exhibit either pro-cancer or anti-cancer role by directly binding to PIWI proteins, highly conserved RNA-binding proteins [10]. In humans, so far four homologs have been identifed: PIWIL1, PIWIL2, PIWIL3, and PIWIL4. PiRNA can influence diverse processes in cancer cells, including apoptosis, proliferation, migration and invasion, by regulating gene expression at both transcriptional and post-transcriptional levels [12–15]. Therefore, the aim of this study was to explore the effect and exact mechanism of piRNA in CC.

In recent years, emerging reports have indicated that epigenetic regulation plays a crucial role in the biological behaviors of cancer [16, 17]. N6-methyladenosine (m6A) of RNA, one of the most prevalent internal epigenetic regulations, has been involved in physiological and pathological processes of CC [18–20]. m6A RNA regulatory proteins can be divided into three classes: methyltransferases, demethylases and m6A-binding proteins. The methyltransferases, also known as writers and responsible for m6A formation, are composed mainly of methyltransferase-like 3 (METTL3), METTL14 and Wilms’ tumor 1-associating protein (WTAP); demethylases, also known as erasers, catalyse the demethylation of m6A and are composed of fat mass and obesity-associated (FTO) and alkB homolog 5 (ALKBH5); m6A-binding proteins, also known as readers and determining the destiny of targeted RNAs, consists primarily of YT521-B homology (YTH) domain family proteins (YTHDF1/2/3) and YTH domain containing proteins (YTHDC1/2) [21–23]. The relationship between m6A RNA and piRNA have been proved in cardiac hypertrophy [24] and diffuse large B cell lymphoma [25].

However, it is unclear whether there is a relation exists between m6A RNA and piRNA in CC. Therefore, we aimed to investigate the expression level of piRNA-14633 in CC and its correlation with prognosis to further explore its role in epitranscriptomic regulation in CC and its potential contribution to CC pathogenesis.

## Materials and methods

### Patients and samples

Formalin-fixed paraffin-embedded cervical cancer samples were obtained from the department of Pathology, Henan Province People's hospital. All patients did not receive radiotherapy, chemotherapy, targeted therapy and immunotherapy before surgery. All patients were diagnosed with colon cancer by HE (Hematoxylin–Eosin) staining. The research was approved by the Medical Ethics Committees of Henan Province People's hospital.

### RNA isolation, sequencing, and data analysis

RNA from individual samples was isolated using the Universal RNA Kit (QIAGEN) and quantified using a Nanodrop spectrophotometer (Thermo Scientific, Waltham, MA, USA). All RNA samples were subjected to RNA integrity analysis using a Bio-Analyzer (Agilent Technologies, Santa Clara, CA, USA). Library preparation from polyA selected RNA and RNA-Seq was carried out by the DNA Sequencing. DESeq2 offers a comprehensive and general solution for gene-level analysis of RNA-seq data. Differentially expressed genes that were identified by both DESeq2.

### Cell culture

Two cervical cancer cell lines Caski and Siha, as well as normal cervical endometrial cell lines End1/E6E7 and HUCEC, were purchased from Wuhan vector science Co.,Ltd (Dong Hu new technology development zone, Wuhan, Hubei, China). Dulbecco Modified Eagle’s Medium (DMEM; Hyclone, South Logan, UT, USA) was employed to culture End1/E6E7 and HUCEC cells, while Caski and Siha cells were cultured in RPMI1640 medium containing at 37 °C in a humidified atmosphere of 5% CO_2_. All the mediums were contained with 10% fetal bovine serum (FBS; Hyclone, South Logan, UT, USA) and and 50 U/ml of penicillin/streptomycin.

### Vectors and transfection

The piRNA-14633 mimic and the piRNA-14633 inhibitor oligonucleotides, as well as the corresponding negative control, were synthesized from Ribobio (Guangzhou, China). Caski and Siha cells were seeded in 6-well plate and cultured overnight for transfection. In accordance with the manufacturer’s instructions, Lipofectamine 2000 (Invitrogen, Carlsbad, CA, USA) and equal amounts of oligo fragments were diluted by Opti-MEM/Reduced serum medium (Thermo Fisher Scientific, Waltham, MA, USA), respectively. Once the two solutions were mixed, the mixture was added into a 6-well plate. The medium was renewed 12 h after transfection. The cells were harvested 48 h after transfection.

### siRNA transfections

Cells were transfected with pools of scrambled or target gene-specific siRNAs (100 nM) using Lipofectamine 2000 according to the manufacturer’s instructions. The sequences of the siRNA against METTL14#1 is as follow: GCAGCACCUCGA UCAUUUATT and the sequence of METTL14#2 is as follow:GGAUGAAGGAGAGACAGAUTT.

The sequence of si-NC is as follow: UUCUCCGAACG UGUCACGUTT.

### RNA m6A quantification

Total RNA was extracted via TRIzol (Invitrogen, CA, USA) as described below, and RNA quality was assessed by NanoDrop (Thermo Fisher Scientific, Waltham, MA, USA). The m6A modification level of total RNA was examined via EpiQuik m6A RNA Methylation Quantification Kit (P-9005; Epigentek Group Inc., Farmingdale, NY, USA) according to the instruction. Briefly, 200 ng RNA accompanied with m6A standard were coated on assay wells, followed by capture antibody solution and detection antibody solution. The m6A levels were quantified colorimetrically by reading the absorbance of each well at a wavelength of 450 nm (OD450), and then calculations were performed based on the standard curve.

### RT-qPCR analysis and RNA stability assay

Total RNA from cell lines and clinical tissues was extracted with Trizol reagent (Invitrogen), the reverse transcription reactions were conducted with oligo (dT) or specific miRNA/piRNA stem-loop RT primers using the Revert Aid First Strand cDNA Synthesis Kit (Thermo). Relative RNA levels determined by RT-qPCR were measured on the Light Cycler 480 II using the SYBR Green method. The primer sequences used are shown in Additional file 1: Table S1. For quantification of piRNAs, real-time qPCR were performed using the Bulge-LoopTM qPCR kit (RiboBio, Guangzhou, China) according to the manufacturer’s protocol. U6 small nuclear RNA was employed as an internal control in both cell lines. All experiments were performed in three biological replicates. The relative expression of RNA was calculated as the power value (2-^△△Ct^). For RNA stability assay: the cells were treated with a-Amanitin at 5 μg/ml. After incubation for 0 h, 12 h, 4 h and 36 h, the cells were collected and RNA was extracted for RT-qPCR described above.

### Cell viability and colony formation assays

Cell viability was measured using the CCK-8 kit. The cells were seeded in the 6-well culture dishes and incubated at 37 °C in humidified incubator for two weeks. Colonies were fixed and stained with crystal violet and the number of colonies was counted.

### Cell cycle and apoptosis assays

The Cell Cycle Analysis Kit (Beijing 4A Biotech Co., FXP0311-100) and AnnexinV/PI apoptosis Kit (Beijing 4A Biotech Co., FXP018-100) were used to assess cell cycle distribution and apoptosis, respectively.

### Wound healing assay

Caski and Siha cells were seeded in the 6-well plate 5 × 10^5^/well so as to form a cell monolayer overnight. The “wound line” was carefully created by a 200 μL sterile plastic tip. Cells were cultured for 24 h. Scratch-wound images at 0 and 48 h were captured using an Olympus IX71 microscope and wound-healing ability was calculated based on the relative cell-free area normalized to that in the 0 h image.

### The invasion and migration assays

Invasion assay was done in a 24-well Millicell chamber. The 8-μm pore inserts were coated with 30 μg of Matrigel (BD Biosciences). Cells were added to coated filters in 200 μL of serum-free medium in triplicate wells. 500 μL medium containing 20% fetal bovine serum was added to the lower chamber as chemoattractant. After 48 h at 37 °C in a 5% CO_2_ incubator, the Matrigel coating on the upper surface of the filter was removed. Cells that migrated through the filters were fixed with methanol, stained with 0.5% crystal violet, and photographed. Cell number on three random fields was counted. The migration assay was conducted in a similar fashion without coating with Matrigel.

### Western blot analysis

Protein extracts from cells were prepared using detergent-containing lysis buffer. Total protein (50 µg) was subjected to SDS-PAGE and transferred to PVDF membrane (Millipore). Antibodies against METTL14 (ab252562), PIWIL2 (ab151398) and PIWIL4 (ab111714) were from Abcam. Antibody against PIWIL1 (15,659–1-AP), CYP1B1 (18,505–1-AP) and β-actin (60,008–1-Ig) were from Proteintech. Antibody against PIWIL3 (sc-398779) was from Santa. Then, the anti-rabbit or mouse horseradish peroxidase (HRP)-conjugated secondary antibody were conducted to incubate the membranes overnight at 4 °C and visualized with Immobilon Western Chemiluminescent HRP Substrate (Millipore).

### Luciferase reporter assay

The METTL14 wild type (METTL14-WT) and METTL14 mutated type (METTL14-MUT) were constructed into luciferase pmirGLO reporter vector and followed by Dual-Glo Luciferase Assay system (NO. E608001, Sangon Biotech Co., Ltd. Shanghai, China). After 36 h transfection, the cells were lysed by passivelysis buffer. Firefly Luciferase (F-luc) and Renilla Luciferase(R-luc) of lysis were detected respectively.

### Animal experiments

Animal experiments were carried out in compliance with approved protocols and guidelines from the Institutional Animal Care and Use Committee. To examine the effects of piRNA-14633 on subcutaneous xenograft growth, BALB/c nude mice (Beijing Vital River Laboratory Animal Technology) were subcutaneously injected with 0.1 mL of cell suspension containing 2 × 10^6^ cells. Tumor volume (mm^3^) was measured every 4 days using a Vernier caliper and calculated as 0.4 x (short length)^2^ × long length. Treatment was initiated when tumors reached a volume of 45–55 mm^3^ (day 16). The mice were randomly divided into 4 groups and received mi-NC, si-NC, mi-14633 and in-14633 by intratumor injection for 20 days. The mice were sacrificed, and the experiment was terminated at the end of 36 days. Tumors were isolated, weighed, and imaged.

### Statistical analysis

All the experiments were performed 3 times at least. SPSS software (version 19.0, IBM Corp., Armonk, NY, USA) was used for statistical analysis of all the experimental data. GraphPad Prism (version 7, GraphPad Software, La Jolla, CA, USA) was used to determine the statistical results. All data are expressed as the mean ± standard deviation (mean ± SD). The statistical analysis of the data from 2 groups was performed using a t-test. The comparisons of multiple groups were performed by one-way ANOVA and then an LSD-t test. P < 0.05 was considered to be significant.

## Results

### piRNA-14633 is overexpressed in cervical cancer

**Fig. 1:**
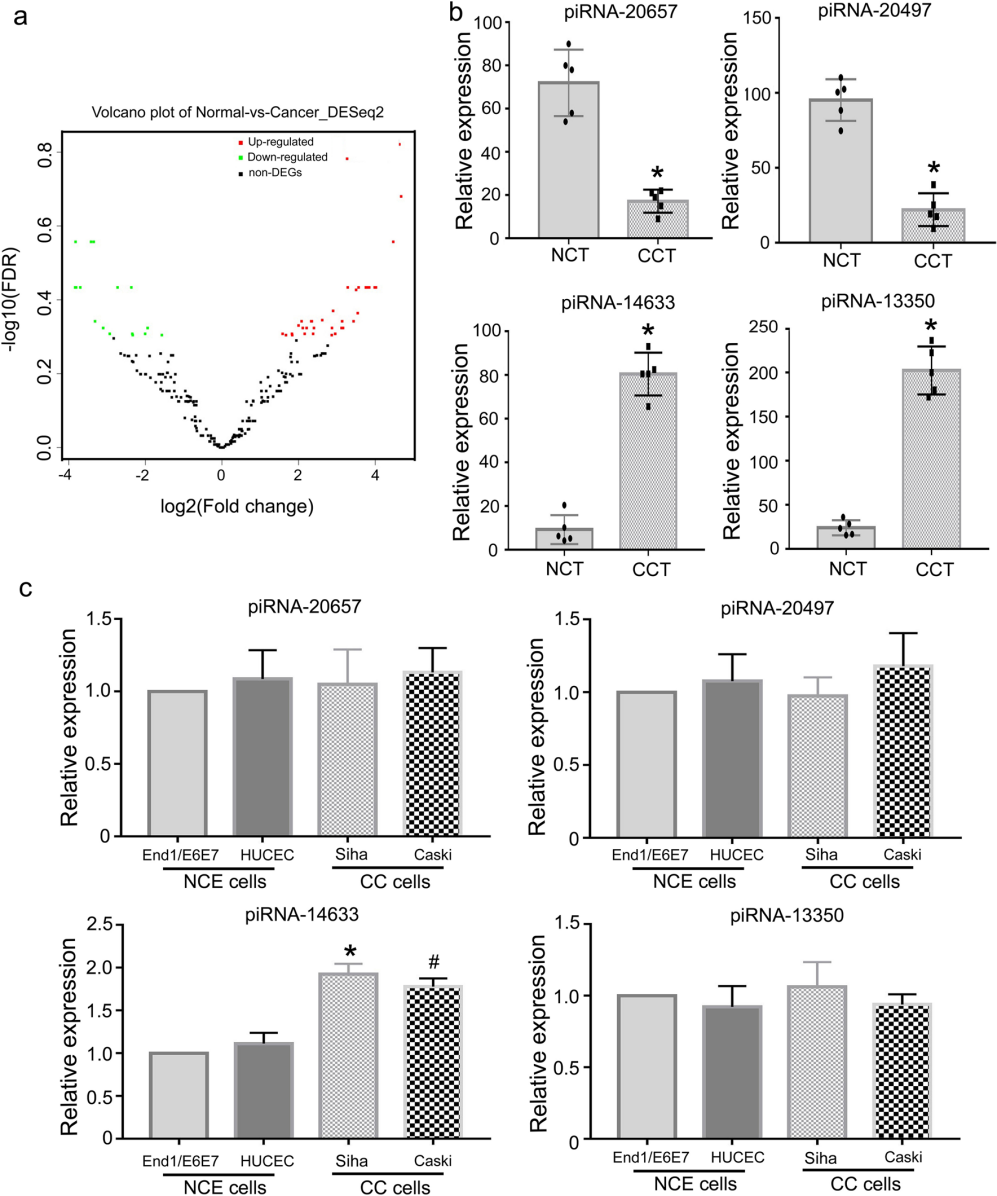
piRNA-14633 is overexpressed in cervical cancer tissue and cell. **a** Folding changes and dispersion of piRNA in NCT and CCT were estimated using the DESeq method. **b** Four piRNAs expressions were compared in NCT and CCT. Data are presented as mean ± SD. *P < *0.05* vs. NCT. **c** Real-time PCR of four piRNAs expressions in CC cells (Siha and Caski) compared with NCE cells (End1/E6E7 and HUCEC). *P < *0.05* vs. NCEC (End1/E6E7 and HUCEC). ^#^*P* < *0.05* vs. NCEC (End1/E6E7 and HUCEC). All experiments are expressed mean ± SD. NCT normal cervical tissue, CCT cervical cancer tissue, NCEC normal cervical endometrial cells, CCC cervical cancer cells

In order to systematically study the role of piRNAs in cervical cancer, we first explored the expression profiles of five pairs of FFPE cervical cancer tissues and normal cervical tissues using the DESeq2 package and found some significant differentially expressed piRNAs with log10 (FDR) > 0.2 in cervical cancer tissues and normal cervical tissue (Fig. 1a). Figure 1b shows four significant differentially expressed piRNAs in cervical carcinoma tissue and normal cervical tissue. piRNA-14633/piRNA-13350 were up-regulated and piRNA-20657/piRNA-20497 were down-regulated in cervical carcinoma tissue compared with normal cervical tissue. Next, we validated the expression of these four piRNAs in normal cervical epithelial cells and cervical cancer cells by RT-PCR. We found that piRNA-14633 was significantly increased in cervical carcinoma cells compared to normal cervical epithelial cells (Fig. 1c). However, no significant difference was found in the expression of the other three piRNAs between cervical carcinoma cells and normal cervical epithelial cells. These results illustrate that piRNA-14633 may be a tumor promoter in cervical carcinoma.

The expression of piRNA-14633 was next explored to see if it was associated with the clinicopathological characteristics of the 94 cervical cancer patients. According to the results of RT-PCR, the 94 cervical carcinoma cases were divided into a piRNA-14633 low expression group (n = 44) and a piRNA-14633 high expression group (n = 50). We found that increased levels of piRNA-14633 were closely related to advanced TNM stage and increased tumor size (Table 1).

**Table 1:** Correlation between the clinicopathological traits and piRNA-14633 expression in cervical carcinoma patients

	Total	n%		P
		Low expression	High expression	
		of piRNA-14633	of piRNA-14633	
Age				
≥ 55 (years)	55	25 (45.5%)	30 (54.5%)	0.755
< 55 (years)	39	19 (48.7%)	20 (51.3%)	
T stage				
T1-T2	49	30 (61.2%)	19 (38.8%)	0.003*
T3-T4	45	14 (31.1%)	31 (68.9%)	
N stage				
N1	42	27 (64.3%)	15 (35.7%)	0.002*
N2	52	17 (32.7%)	35 (67.3%)	
M stage				
M0	34	22 (64.7%)	12 (35.3%)	0.009*
M1	60	22 (36.7%)	38 (63.3%)	
Tumor size (cm)				
< 4	41	25 (61.0%)	16 (39.0%)	0.015*
≥ 4	53	19 (35.8%)	34 (64.2%)	

T stage: Primary tumor stage; N stage: Regional lymph nodes stages; M stage: Distant metastasis stage

### piRNA-14633 promotes proliferation ability of cervical carcinoma cells

**Fig. 2:**
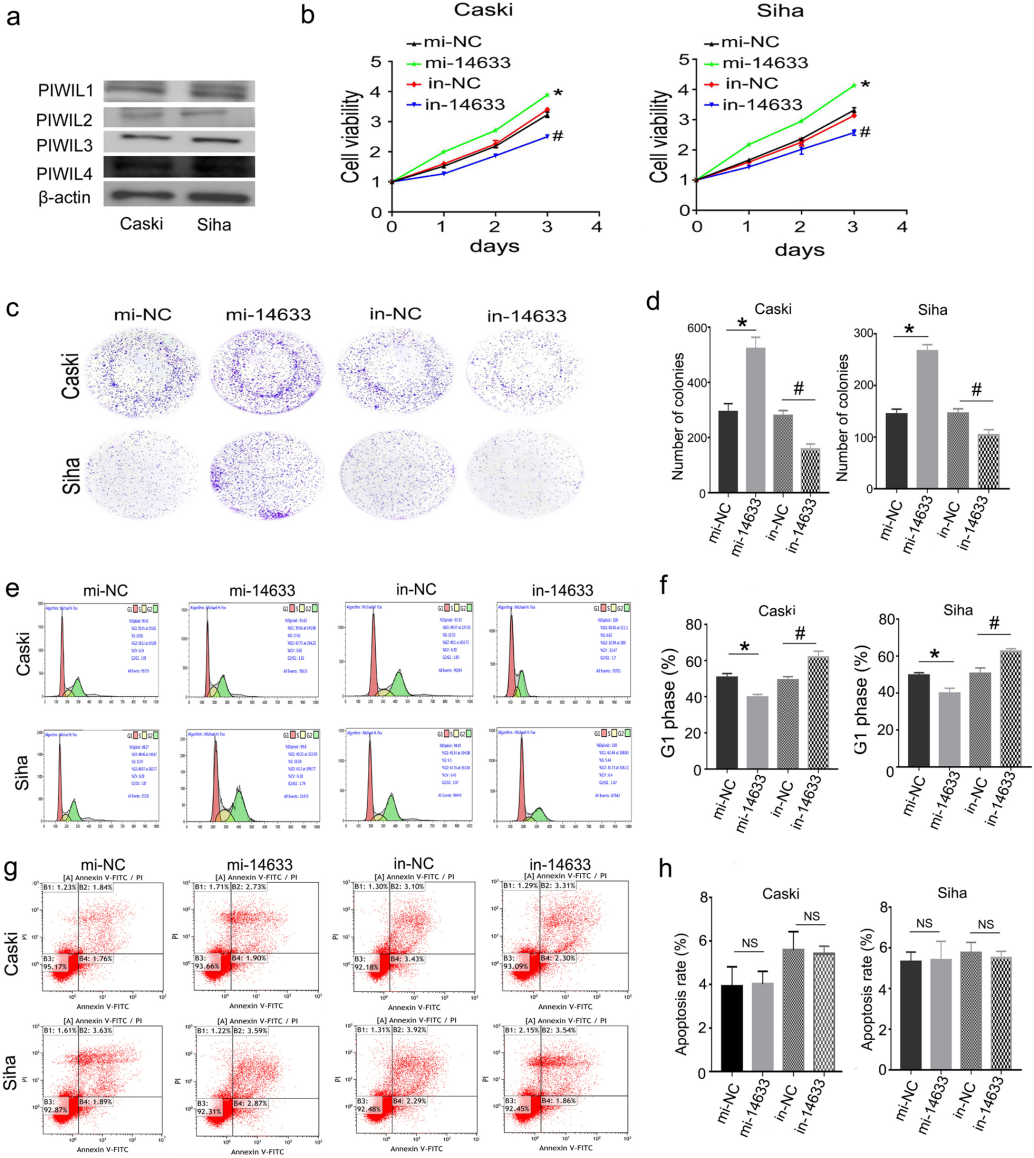
piRNA-14633 promotes proliferation in Siha and Caski cells. **a** Western blot was performed to detect the expression of PIWIL1, 2, 3 and 4 in CC cells. **b** CCK-8 assay was used to assess cell viability in cell treated with 50 nM mi-14633 or 50 nM in-14633. **c**, **d** Colony formation assay was used to assess cell proliferation. **e**, **f** Flow cytometry was used to analyze cell cycle. **g**, **h** Flow cytometry was used to analyze cell apoptosis. *P < *0.05* vs. mi-NC group. ^#^*P* < *0.05* vs. in-NC group. mi-14633 piRNA-14633 mimic, in-14633 piRNA-14633 inhibitor, NS not significant

Prior to studying possible roles of piR-14633, we investigated the expression of PIWIL1, 2, 3 and 4 in Caski and Siha cells, which are the prime proteins for biogenesis and functions of piRNAs. The results indicated that four homologs were expressed in CC cells (Fig. 2a),indicating their association with piRNA biogenesis and piRNA mediated regulatory functions. Next,we interfered with piRNA-14633 expression in Caski and Siha cells with mimic or inhibitor to to analyze its biological functions. We depleted piRNA-14633 expression using piRNA-14633 inhibitor and increased piRNA-14633 expression using piRNA-14633 mimic in Caski and Siha cells. The efficiency of transfection was examined by RT-PCR. The results showed that the piRNA-14633 inhibitor reduced its expression and the piRNA-14633 mimic increased its expression in Caski and Siha cells (Fig. S1). As shown in CCK-8, inhibition of piRNA-14633 expression resulted in a dramatic decrease in the proliferation rate of Caski and SiHa cells, while increasing piRNA-14633 expression increased the proliferation rate of both cells (Fig. 2b). Similarly, the colony formation assay confirmed that piRNA-14633 increased the number of Caski and SiHa colonies (Fig. 2c and d).

Cycle and apoptosis of Caski and SiHa cells were examined by flow cytometry. As shown in Fig. 2e and f, inhibiting piRNA-14633 promoted cell cycle arrest in the G1 phase, and piRNA-14633 overexpression inhibited cell cycle arrest in the G1 phase. No significant apoptotic changes, however, were observed in Caski and SiHa cells for piRNA-14633 inhibitor or piRNA-14633 mimic compared to negative control for inhibitor or negative control for mimic, respectively (Fig. 2g and h). The above results indicated that piRNA-14633 promoted proliferation ability and had no effects on apoptosis of cervical carcinoma cells.

### piRNA-14633 promotes migration/invasion abilities of cervical carcinoma cells

**Fig. 3:**
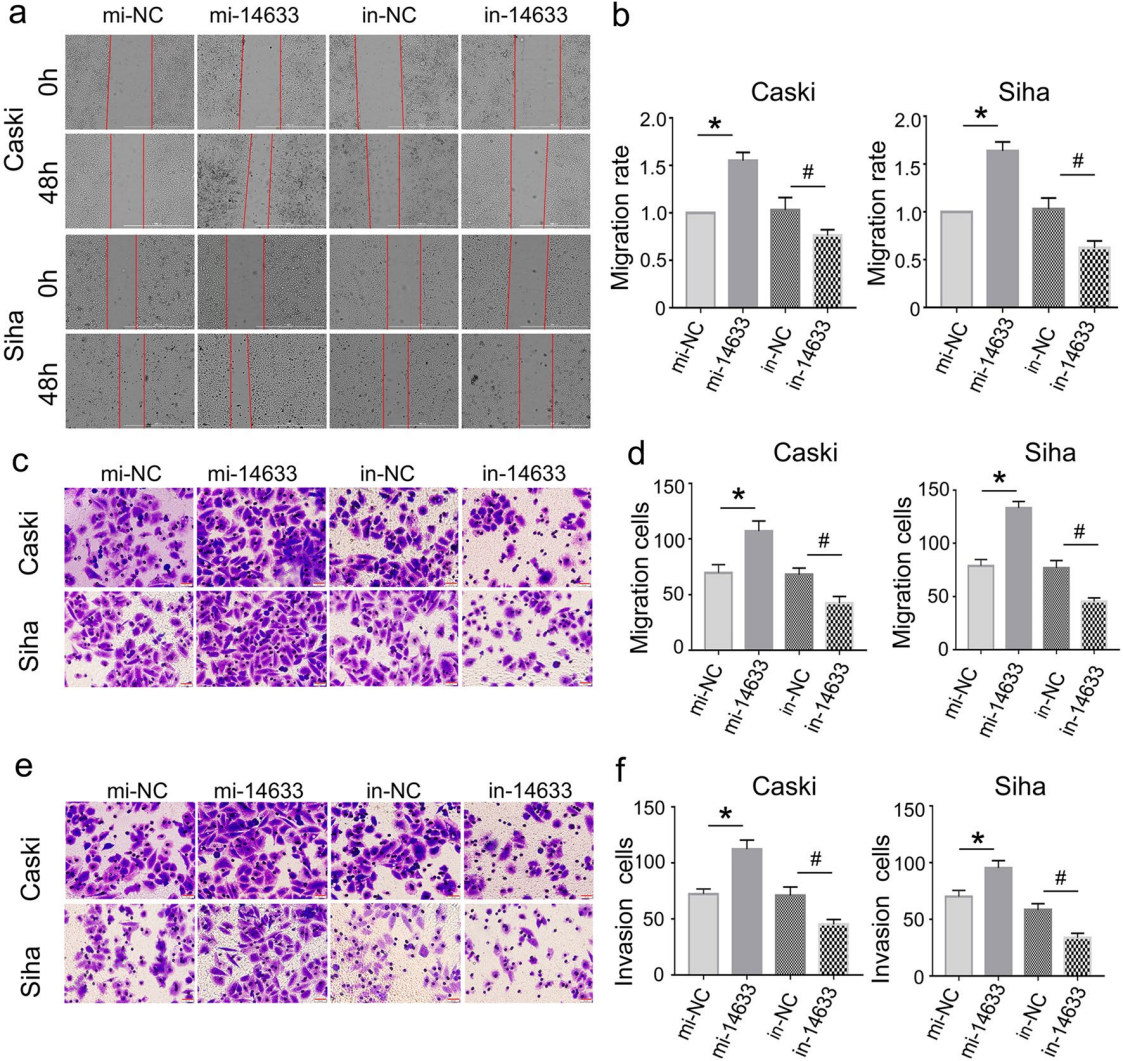
piRNA-14633 accelerates migration/invasion abilities of Siha and Caski cells treated with 50 nM mi-14633 or 50 nM in-14633. **a**, **b** Wound healing assay was used to analyze the migration capabilities of two cervical cancer cells. Scale bars, 1000 μm. **c**, **d** Transwell assay was applied to measure migration abilities of Siha and Caski cells. Scale bars, 20 μm. **e**, **f** Transwell assay was applied to measure invasion abilities of Siha and Caski cells. Scale bars, 20 μm. *P < 0.05 vs. mi-NC group. ^#^*P* < *0.05* vs. in-NC group. mi-14633 piRNA-14633 mimic, in-14633 piRNA-14633 inhibitor

Cell invasion and migration are known as the main indicators for assessing metastatic capacity. In contrast to the negative control inhibitor group, the trabecular surface of Caski and SiHa cells in the piRNA-14633 inhibitor group became significantly wider and migration weaker, as shown in Fig. 3a and b. While overexpression of piRNA-14633 induced narrower wound field of Caski and SiHa cells. Consistently, transwell results showed that piRNA-14633 overexpression increased the migration abilities of both cells and piRNA-14633 knockdown inhibited the migration abilities of both cells (Fig. 3c and d). We then tested the invasion abilities of cervical carcinoma cells by matrigel transwell assay. As shown in Fig. 3e and f, piRNA-14633 overexpression promoted invasion of Caski and SiHa cells compared to the mi-NC group, whilst cell invasion was attenuated in the piRNA-14633 inhibitor group compared to the in-NC group. These results suggest that piRNA-14633 could promote the invasion and migration abilities of cervical carcinoma cells.

### piRNA-14633 up-regulates m6A methylation and increases METTL14 expression in cervical carcinoma cells

**Fig. 4:**
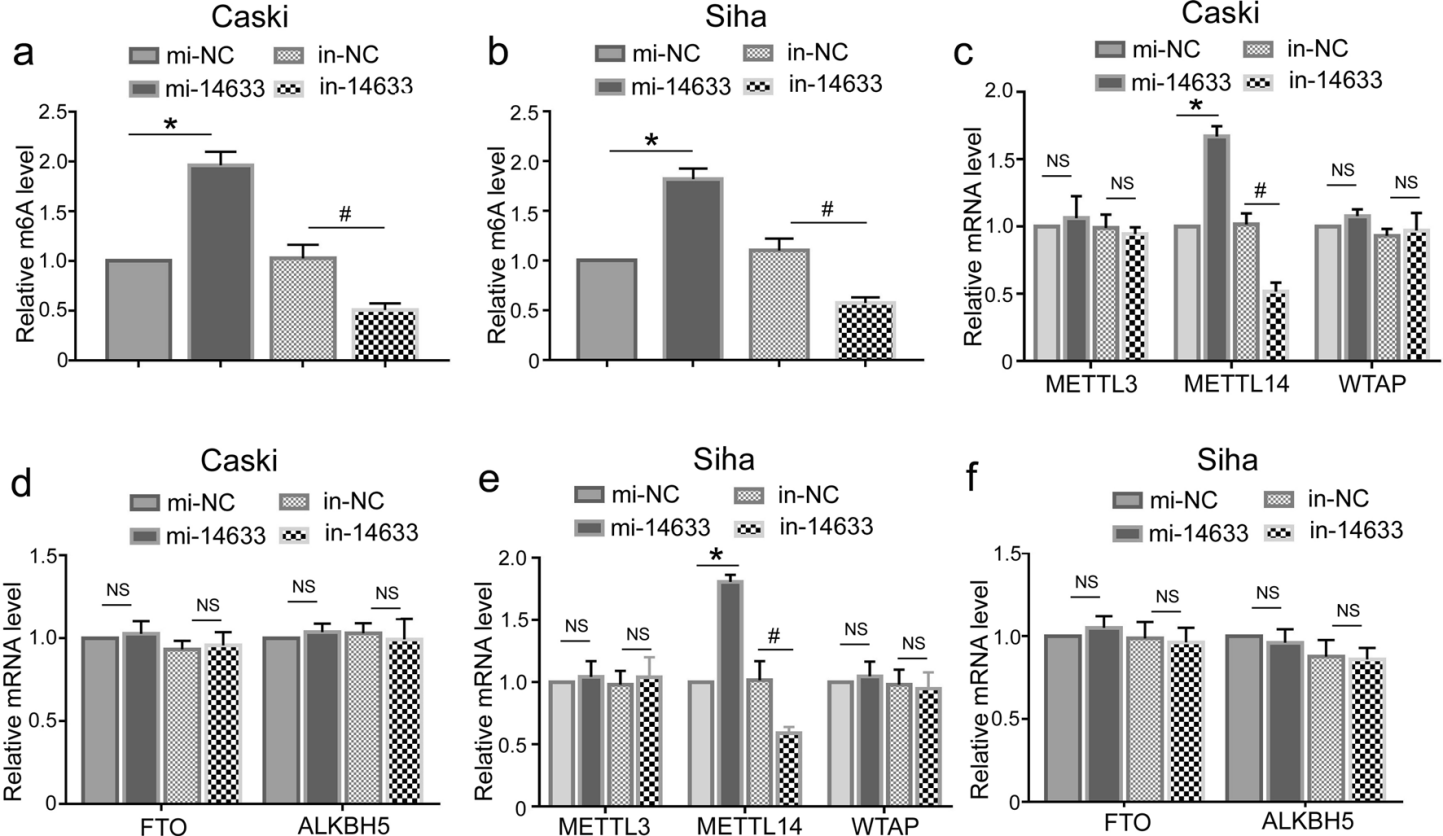
piRNA-14633 increases m6A levels and elevates METTL14 mRNA expression. **a**, **b** EpiQuik M6A RNA Methylation Quantification Kit (colorimetric method) was used to detect m6A levels in Siha and Caski cells treated with 50 nM mi-14633 or 50 nM in-14633. **c**, **d** Real-time PCR was performed to detect METTL3, METTL14, WTAP, ALKBH5 and FTO mRNA levels in Caski cells. **e**, **f** Real-time PCR was performed to determine METTL3, METTL14, WTAP, ALKBH5 and FTO mRNA levels in Siha cells. *P < *0.05* vs. mi-NC group. ^#^*P* < *0.05* vs. in-NC group. mi-14633 piRNA-14633 mimic, in-14633 piRNA-14633 inhibitor

Considering that piRNAs mainly act as important factors in m6A methylation, we first explored whether piRNA-14633 affected the expression level of m6A methylation in cervical carcinoma cells. The results show that the total m6A abundance increased with the overexpression of piRNA-14633 and decreased with the down-regulation of piRNA-14633 (Fig. 4a and b).

RNA methyltransferases/demethylases play a crucial role in m6A methylation. We then examined the expression levels of METTL3, METTL14, WTAP, FTO and ALKBH5 by RT-PCR in Caski and SiHa cells treated with piRNA-14633 mimic or inhibitor. The results showed that silencing piRNA-30473 could reduce the mRNA expression of METTL14, while overexpression of piRNA-30473 increased the mRNA expression of METTL14 (Fig. 4c–f). But the mRNA expressions of other four genes were not significantly different in cells when in response to piRNA-14633 inhibitor or piRNA-14633 mimic, when compared to negtive control inhibitor or negtive control mimic, respectively. These results indicated that piRNA-14633-mediated upregulation of m6A methylation levels might be relevant to to increased METTL14 expression in cervical carcinoma cells.

### METTL14 is a target of piRNA-14633 in cervical carcinoma cells

**Fig. 5:**
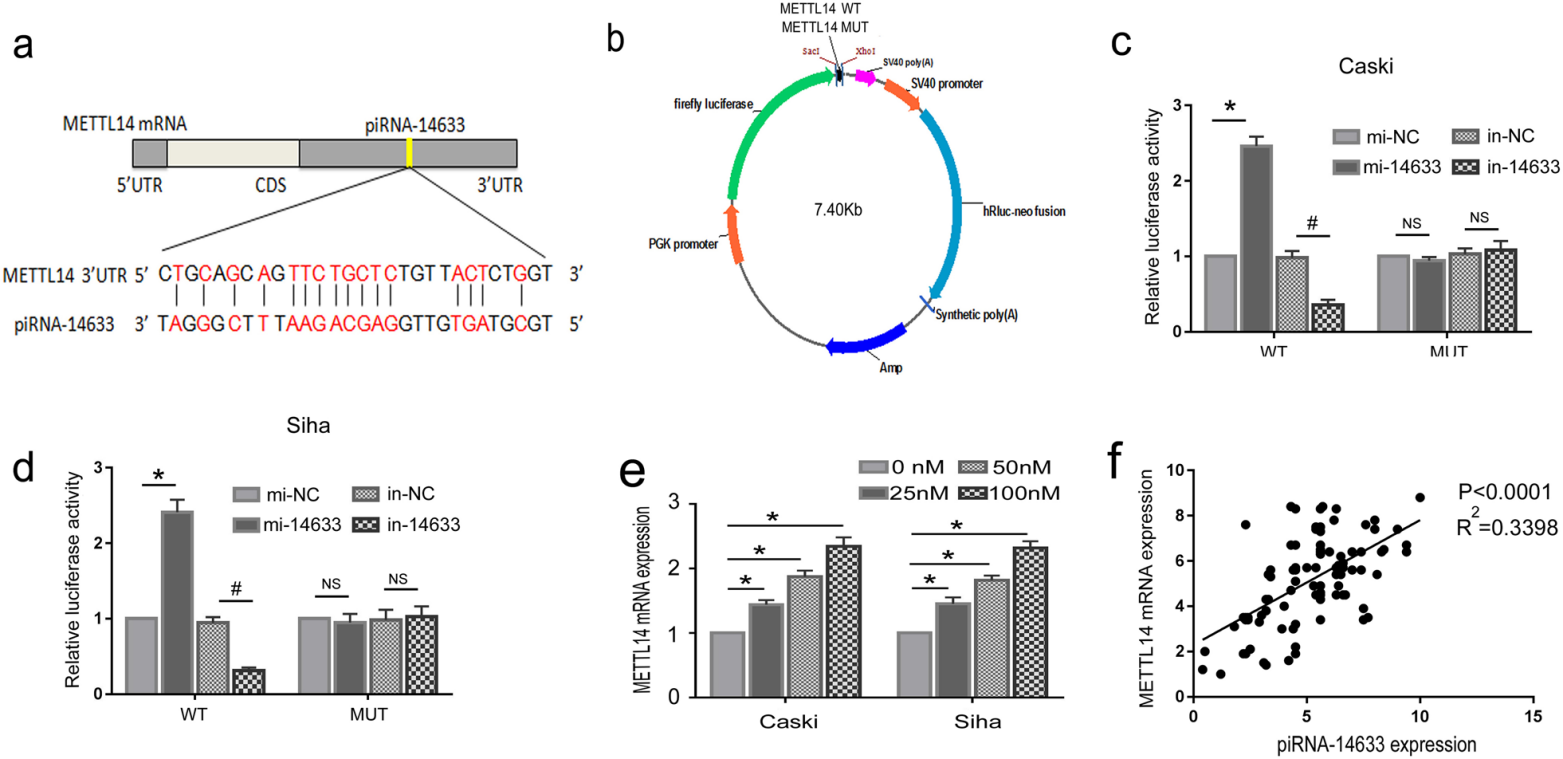
METTL14 is a target of piRNA-14633 in Siha and Caski cells. **a** Diagram of the interaction between piRNA-14633 and the 3’UTR of METTL14. **b** Wild/mutant-type 3’UTR of METTL14 luciferase pmirGLO reporter vector. **c**, **d** Measurement of relative luciferase activity in Siha and Caski cells transfected with METTL14 -wild type or mutant-type, with 50 nM mi-14633 or 50 nM in-14633. **e** Real-time PCR was performed to detect the effect of piRNA-14633 (0 nM, 25 nM, 50 nM and 100 nM) on METTL14 mRNA expression **f** The correlation of piRNA-14633 with METTL14 expression in clinical FFPE specimens. *P < *0.05* vs. mi-NC group. ^#^*P* < *0.05* vs. in-NC group. mi-14633 piRNA-14633 mimic, in-14633 piRNA-14633 inhibitor, NS not significant

To verify whether piRNA-14633 targets METTL14 in cervical carcinoma cells, we predicted the binding site between piRNA-14633 and 3’UTR of METTL14 (mRNA 5'-3', 4364–4392) by bioinformatics software (Fig. 5a). We then performed a dual-luciferase reporter assays by constructing wildtype (WT) and mutant (MUT) dual-luciferase reporter vectors of METTL14 according to the potential binding sites of piRNA-14633 and METTL14. Figure 5b exhibites the wild/mutant-type 3’UTR of METTL14 luciferase pmirGLO reporter vector. Translation of the luciferase reporter gene showed that silencing of piRNA-14633 resulted in a decrease in luciferase activity of the reporter construct carrying WT METTL14 relative to the inhibitor of the negative control, while there was no significant change in luciferase activity of the MUT reporter (Fig. 5c and d). Similarly, piRNA-14633 overexpression caused a increase in the luciferase activity of the reporter construct carrying the WT METTL14 relative to the negtive control minic, whereas the luciferase activity of MUT reporters was not significantly changed (Fig. 5c and d). We also confirmed that the effect of piRNA-14633 on METTL14 mRNA expression has concentration-dependent characteristics (Fig. 5e). In clinical specimens, we found that the expression of piRNA-14633 showed a strong positive correlation with METTL14 mRNA levels (Fig. 5f). The above results indicate that piRNA-14633 binds specifically to METTL14 in cervical carcinoma cells.

### piRNA-14633 elevates METTL14 protein expression by increasing stability of its mRNA in cervical carcinoma cells

**Fig. 6:**
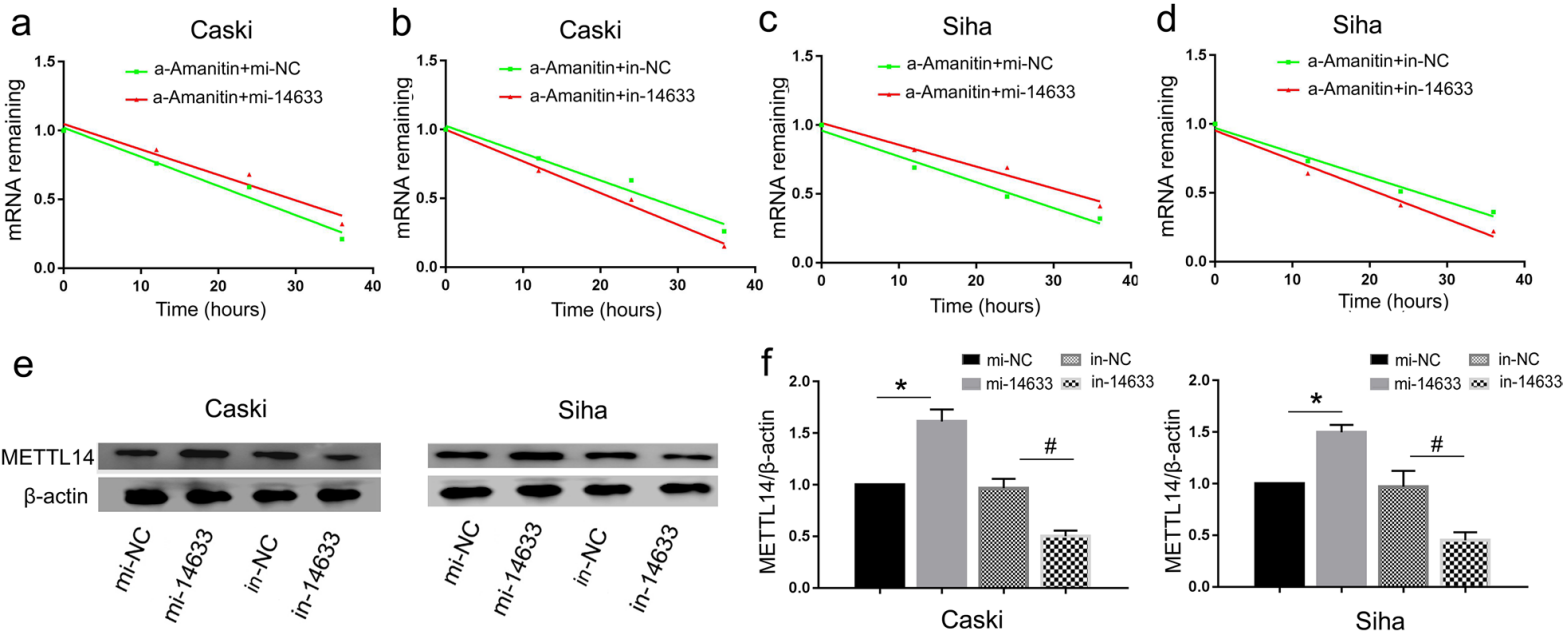
piRNA-14633 increases stability of METTL14 mRNA and elevates METTL14 protein expression. **a**, **b** Real-time PCR was performed to determine METTL14 mRNA levels in Caski cells treated with 50 nM mi-14633 or 50 nM in-14633 and / or a-Amanitin. **c**, **d** Real-time PCR was performed to determine METTL14 mRNA levels in Siha cells treated with altered expression of piRNA-14633 and/or a-Amanitin. **e**, **f** Western blot analysis of the METTL14 expression in Siha and Caski cells. *P < *0.05* vs. mi-NC group. ^#^*P* < *0.05* vs. in-NC group. mi-14633 piRNA-14633 mimic, in-14633 piRNA-14633 inhibitor

To further study the relationship between piRNA-14633 and METTL14 in cervical carcinoma cells, we inhibited new mRNA synthesis of cervical carcinoma cells by a-Amanitin. By RT-PCR, we found that piRNA-14633 overexpression increases half-life of METTL14 and piRNA-14633 knockdown decreases half-life of METTL14 in cervical carcinoma cells (Fig. 6a–d). In the next step, we examined protein expression of METTL14 in Caski and SiHa cells treated with piRNA-14633 overexpression or knockdown. Western blot assays showe that piRNA-14633 overexpression could upregulate the expression of METTL14 (Fig. 6e and f). We also found that piRNA-14633 correlated positively with METTL14 mRNA expression in clinical FFPE specimens (Fig. 6g). These results suggest that piRNA-14633 increases stability of METTL14 mRNA, which then elevated METTL14 protein expression in cervical carcinoma cells.

### piRNA-14633 promotes proliferation ability of cervical carcinoma cells by increasing m6A modification

**Fig. 7:**
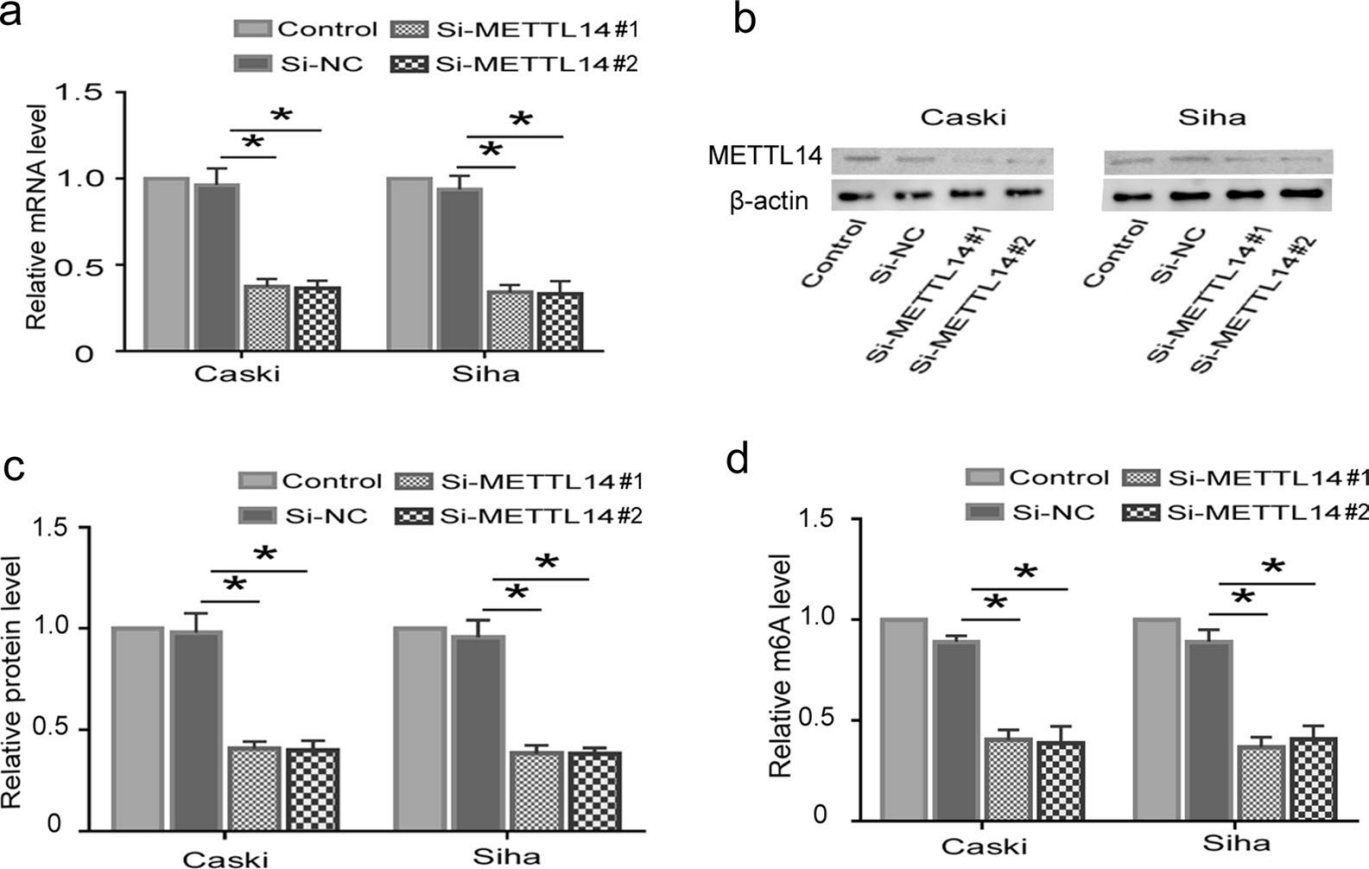
METTL14 knockdown effectively attenuates m6A levels in Siha and Caski cells. **a** Real-time PCR detection of METTL14 expression in Siha and Caski cells after transfection with 50 nM si-NC or 50 nM si-METTL14#1/2. **b**, **c** Western blot was performed to detect the expression of METTL14 in Siha and Caski cells after transfection with si-NC or si-METTL14#1/2. **d** EpiQuik M6A RNA Methylation Quantification Kit (Colorimetric assay) was used to detect m6A levels in Siha and Caski cells. *P < *0.05* vs. si-NC group. Si-NC siRNA-negtive control, si-METTL14#1/2 siRNA-METTL14#1/2

**Fig. 8:**
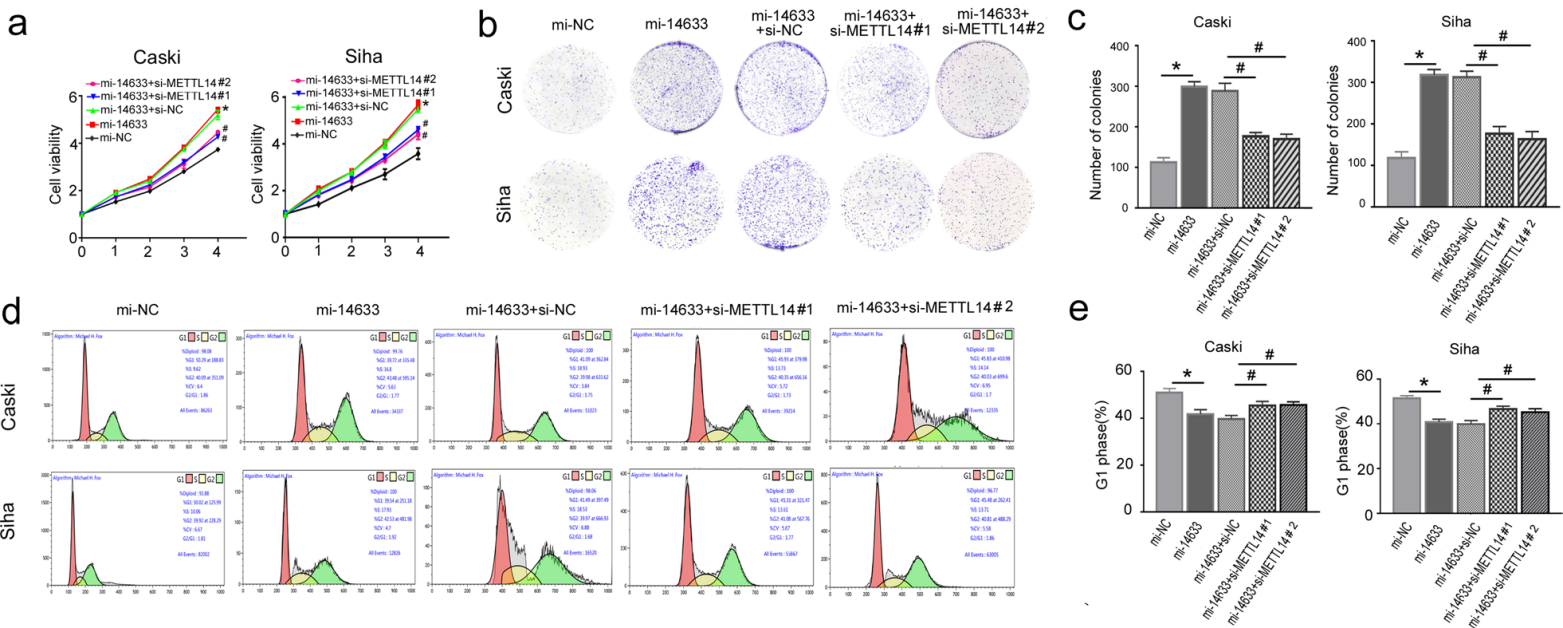
piRNA-14633 promotes proliferation of cervical cancer cells via METTL14 binding. **a** CCK-8 assay was used to assess viability in cells transfected with 50 nM mi-14633 or co-transfected with 50 nM si-METTL14#1/2. **b**, **c** Colony formation assay was used to assess cell proliferation. **d**, **e** Flow cytometry was used to analyze cell cycle. *P < *0.05* vs. mi-NC group. ^#^*P* < *0.05* vs. mi-14633 + si-NC group. mi-14633 piRNA-14633 mimic, in-14633 piRNA-14633 inhibitor, si-NC siRNA-negtive control, si-METTL14#1/2 siRNA-METTL14#1/2

To further study the role of METTL14 in piRNA-14633-mediated cervical cancer cells, we knocked down METTL14 expression using small interference RNA (si-METTL14). RT-PCR and Western blot analysis showed that both si-METTL14 #1 and si-METTL14 #2 reduced its mRNA and protein levels in Caski and Siha cells (Fig. 7a–c). By Epiquik m6A RNA measurement quantifcation kit, we found that si-METTL14 decreased m6A methylation activity in cervical carcinoma cells (Fig. 7d). CCK-8 and colony formation assays showed that piRNA-14633 promoted the proliferation of cervical carcinoma cells and that si-METTL14 could block the promotion effect (Fig. 8a–c). Furthermore, overexpression of piRNA-14633 inhibited cell cycle arrest in the G1 phase, which could be partly reverted by METTL14 silencing (Fig. 8d and e). These results demonstrated that piRNA-14633 could enhance the proliferation ability of cervical carcinoma cells by increasing m6A methylation activity.

### piRNA-14633 promotes migration/invasion abilities of cervical carcinoma cells by METTL14-regulated m6A modification

**Fig. 9:**
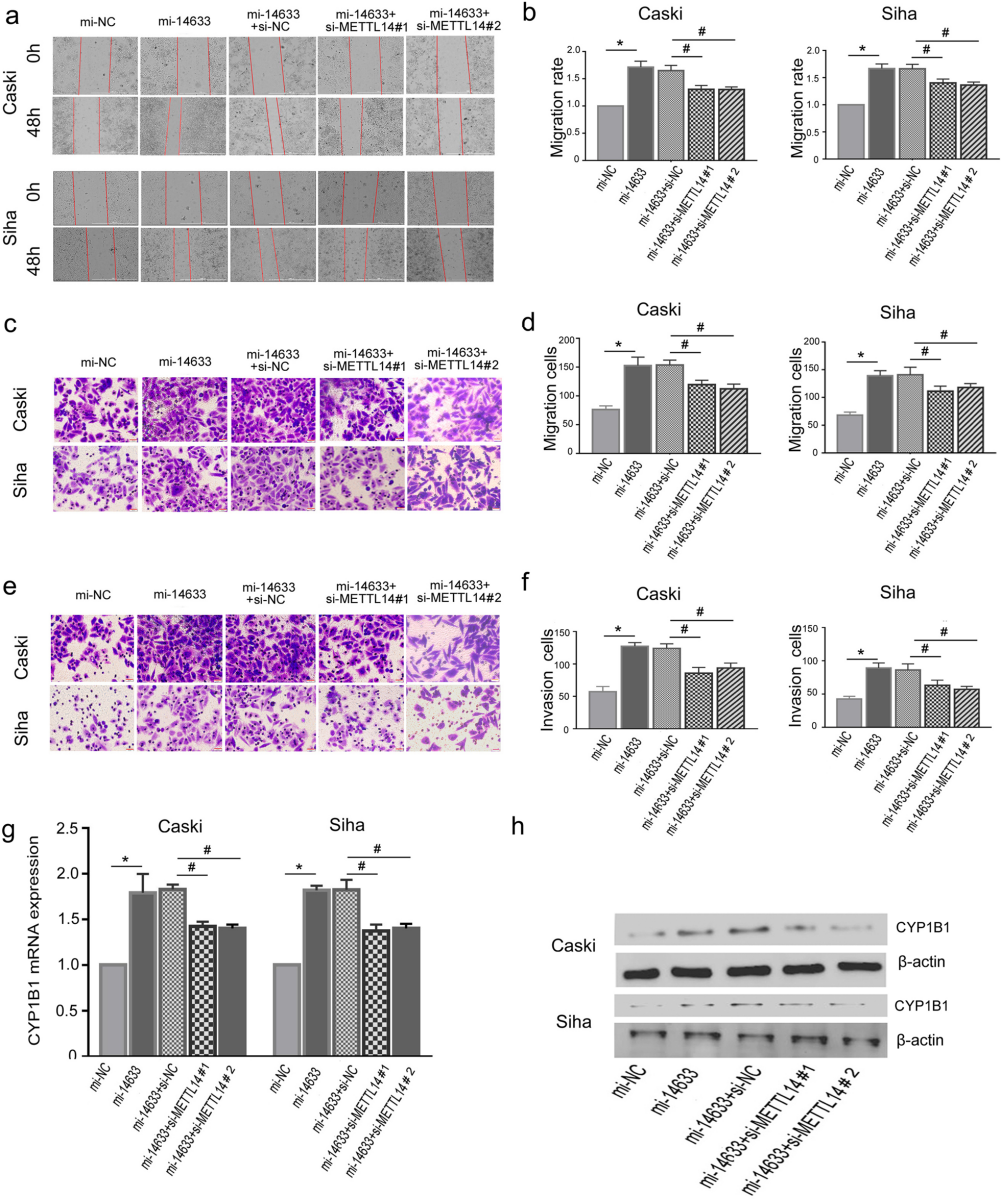
piRNA-14633 contributed to tumorigenesis of Siha and Caski cells via METTL14/CYP1B1 axis. a and b Wound healing assay was used to analyze the migration capabilities of two cervical cancer cells transfected with 50 nM mi-14633 or co-transfected with 50 nM si-METTL14#1/2. Scale bars, 1000 μm. c and d Transwell assay was applied to measure migration abilities of Siha and Caski cells. Scale bars, 20 μm. e and f Transwell assay was applied to measure invasion abilities of Siha and Caski cells. g and h RT-PCR and Western blot waere used to analyze the expressions of CYP1B1 in two cervical cancer cells transfected with 50 nM mi-14633 or co-transfected with 50 nM si-METTL14#1/2. Scale bars, 20 μm. *P < *0.05* vs. mi-NC group. ^#^*P* < *0.05* vs. mi-14633 + si-NC group. mi-14633 piRNA-14633 mimic, in-14633 piRNA-14633 inhibitor, si-NC siRNA-negtive control, si-METTL14#1/2 siRNA-METTL14#1/2

Wound healing assays then showed that piRNA-14633 tended to increase migration of cervical carcinoma cells, which were reversed by si-METTL14#1 and si-METTL14#2 (Fig. 9a and b). We also performed transwell assays and noticed that piRNA-14633 promoted the migration and invasion abilities of cervical carcinoma cells, while silencing of METTL14 impaired these phenotypes (Fig. 9c–f). This results provides some evidence for the existence of piRNA-14633/ METTL14/ m6A modification axis in cervical carcinoma cells.

### CYP1B1 may be target of METTL14-mediated m6A methylation regulated by piRNA-14633

Previous studies have elucidated that CYP1B1 is critical downstream target of METTL14 in caner cells [26]. Of note, the secondary structures of CYP1B1 show a higher confidence m6A methylation sites [26]. We therefore hypothesized that METTL14/ CYP1B1 pathway might be involved in piRNA-14633-induced CC cell metastasis. By RT-PCR and Western blot, we reach the conclusion that piRNA-14633 promotes CYP1B1 expression by METTL14-mediated m6A methylation (Fig. 9g and h), suggesting that CYP1B1 may be a novel candidate direct target of METTL14-mediated m6A methylation regulated by piRNA-14633 in CC cells.

### piRNA-14633 promotes the growth of cervical carcinoma cells in vivo

**Fig. 10:**
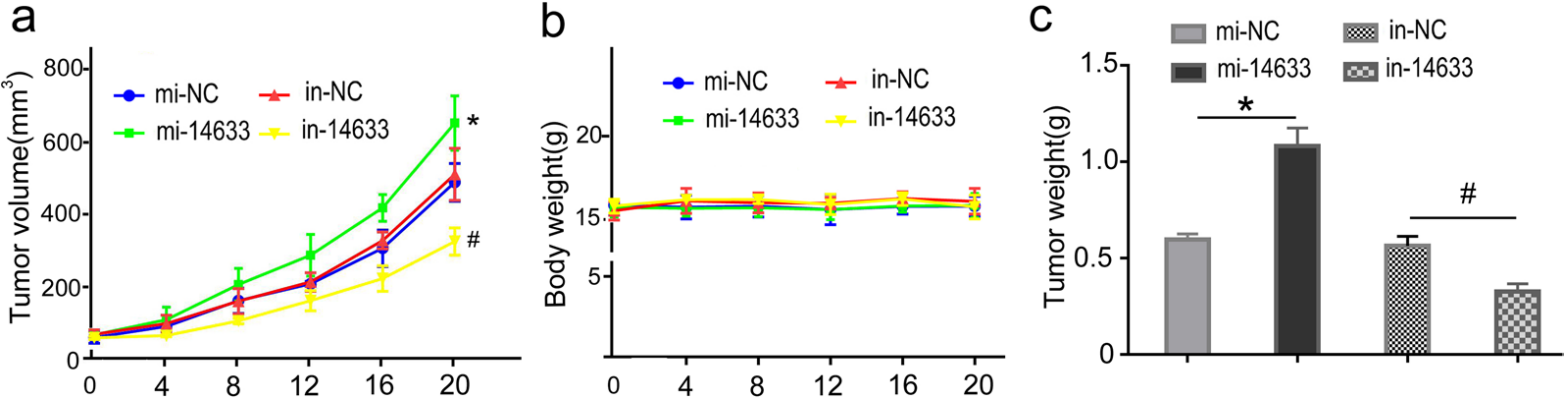
piRNA-14633 promotes the growth of Caski-derived tumor xenografts in Balb/c nude mice. **a** Caski-derived tumors were treated with 50 nM mi-14633 or 50 nM in-14633 for 20 days. The volume of subcutaneous xenografts in nude mice was recorded every four days. **b** Tumor volumes in four different groups. **c** Body weights of mice from four different groups. *P < *0.05* vs. mi-NC group. ^#^*P* < *0.05* vs. mi-14633 + si-NC group. mi-14633 piRNA-14633 mimic, in-14633 piRNA-14633 inhibitor

To further explore the effect of piRNA-14633 on cervical carcinoma in vivo, we a established xenograft mouse model by injecting equal amounts of Caski cells into the nude mice (3 mice in each group). Caski-derived tumors were treated with mi-14633 or in-14633 for 20 days. We then sacrificed the mice and found that piRNA-14633 promoted tumor growths (Fig. 10a). piRNA-14633expression had no impact on body weights of mice (Fig. 10b). The weight of tumors in the mi-NC group were lighter than that in the piRNA-14633 overexpression group (Fig. 10c). Similarly, the tumour weight of the piRNA-14633 knockdown group were lighter than that in the in-NC group (Fig. 10c). The aforementioned findings demonstrate that piRNA-14633 promoted tumor growth in cervical carcinoma.

## Discussion

To date, over 20,000 piRNAs have been identified in eukaryotic genome. Aberrant expression of piRNAs has been demonstrated in a variety of cancers, including diffuse large B-cell lymphoma, colorectal cancer, lung cancer and other cancers[27–30]. Besides, piRNA has been reported to play a protumorigenic or antitumorigenic role by regulating some signaling molecules and signaling pathways[28]. Recent studies have already proved that piRNA-651 is highly expressed in lung cancer and promotes proliferation, invasion and metastasis by regulating expression of caspase-3, Bax and cyclinD 1[31]. piRNA-823 was found by Ding et al. to stimulate the growth of luminal breast cancer cells by regulating cancer stem cells[32]. In this study, we have confirmed the high expression rate of piRNA-14633 in CC cells and tissues.

We also found that overexpression of piRNA-14633 promotes proliferation, migration and invasion of Caski and Siha cells. piRNA-14633 overexpression induced G2/M arrest, regardless of apoptosis. Contrastingly, down-regulation of piRNA-14633 inhibits cells proliferation, migration and invasion, as well as induces G1 arrest. Using a subcutaneous tumour model, we demonstrated that piRNA-14633 contributes to the growth of the CC in vivo. The results illustrate that piRNA-14633 may be a new regulator and a novel target for CC intervention and treatment.

The piRNA was indicated to be significantly associated with DNA methylation in the mammalian cells [33–35]. Specifically, through a proteomic approach, Zoch et al. reported that piRNA is critically involved in DNA methylation of the transposable elements by regulating SPOCD1 expression [34]. piRNA was also shown by Fu et al. to have the ability to promote DNA methylation of genes encoding non-translocatable related proteins [33]. In addition to their involvement in DNA methylation, piRNA has also been reported to be closely related to RNA methylation [36]. Cardiac hypertrophy-associated piRNAs have been shown to induce cardiomyocyte hypertrophy and poor cardiac remodeling by blocking m6A RNA methylation of PARP10 [24]. Han et al. proved that piRNA-30473 contributes to the progression of diffuse large B-cell lymphomaby increasing WTAP mRNA stability and enhancing HK2 m6A level [25]. In recent years, there have been many reports about the role of m6A RNA methylation in CC development and progression [20, 37, 38]. Ma et al. systematically analyzed the expression and clinical prognostic role of 13 m6A RNA methylation regulators in CC by cancer genome mapping [39]. Zou et al. showed that FTO contributes to proliferation and migration of CC and the cancer-promoting function of FTO in CC is dependent on m6A RNA demethylase [40]. In addition, increased METTL3 levels has been found in CC tissue and accelerated the CC carcinogenesis and aerobic glycolysis via up-regulating m6A RNA content [18]. However, there are no reports on relationship of piRNA expression and m6A RNA in CC.

Here, we identified that piRNA-14633 increases m6A RNA methylation in Caski and Siha cells. By bioinformation analysis and dual-luciferase reporter assays, we confirmed that piRNA-14633 specifically targets the 3’-UTR of METTL14. Besides, it was found that piRNA-14633 increased the expression of METTL14 by enhancing its mRNA stability. In clinical specimens, the expression of piRNA-14633 showed a strong positive correlation with METTL14 mRNA levels. To further demonstrate the role of METTL14 in m6A RNA methylation and tumorigenicity of CC, we decreased METTL14 expression by siRNA technology. Down-regulation of METTL14 weakened m6A RNA methylation levels induced by piRNA-14633. Si-METTL14 could counteract the promoting effect of piRNA-14633 on the proliferation, migration and invasion of cervical cancer cells, suggesting that piRNA-14633 accelerates cervical cancer progression by inducing METTL14-mediated m6A methylation. Of course, more work needs to be done to identify m6A reader protein and METTL14 mediated m6A methylated target gene transcripts in piRNA-14633-treated CC cells. Studies have reported that CYP1B1 was expressed in the majority of the cervical cancer samples (91/100, 91.0%) but not in normal healthy cervical samples [41]. Our work speculates a unrecognized piRNA-14633-METTL14-CYP1B1 signaling axis in CC cells. By directly binding to METTL14, piRNA-14633 targets METTL14 through the specific binding domain and subsequently stabilizing the expression and its downstream target such as CYP1B1. Hence, we therefore hypothesized that the METTL14/CYP1B1 pathway might be involved in piR-14633-induced CC cell metastasis.

**Fig. 11:**
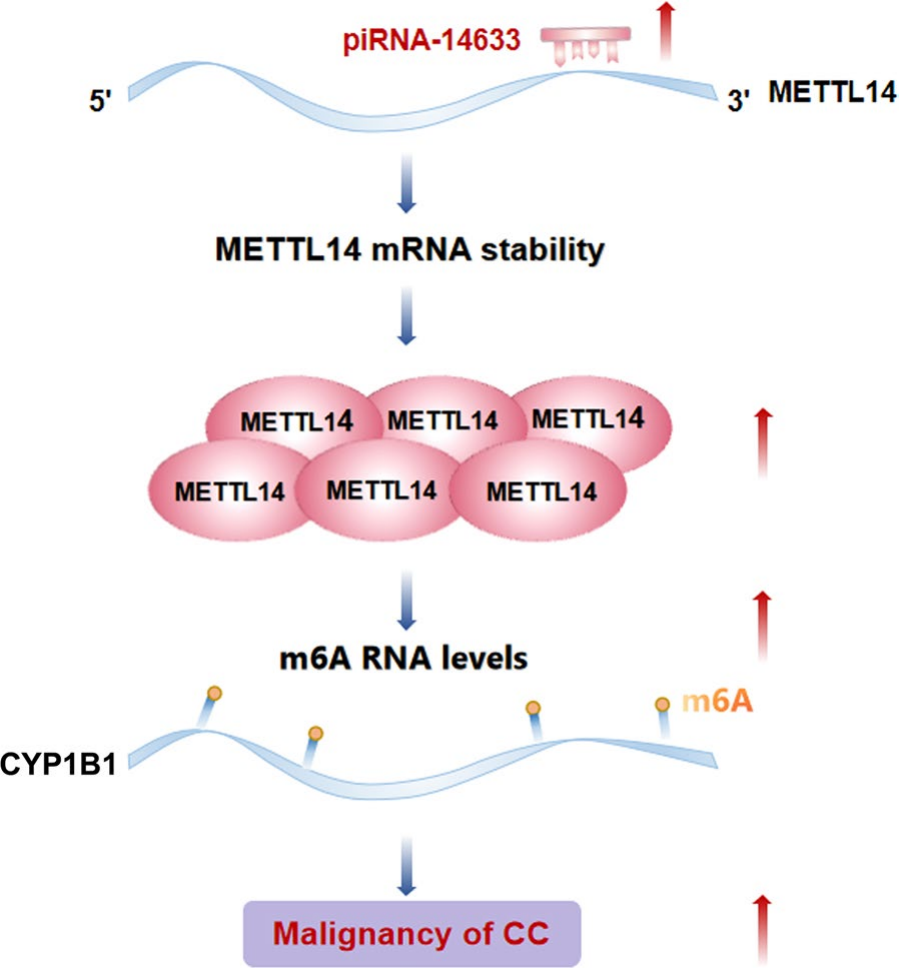
piRNA-14633 promotes CC progression by way of m6A RNA methylation. piRNA-14633 is overexpressed in cervical cancer. piRNA-14633 interactes with 3’UTR of METTL14 to increase METTL14 mRNA stability and promoted the methylase activity of METTL14 promoting the m6 A methylation levels of the downstream target (CYP1B1) and subsequently enhanced expression of CYP1B1, thereby contributed to tumorigenesis of CC

In conclusion, recent studies not only reported higher levels of piRNA-14633 in CC than in normal tissues, but also revealed that piRNA-14633 increased m6A RNA methylation and thus contributed to tumorigenicity of CC by specifically targetting the 3’-UTR of METTL14 and thereby triggering corresponding signaling cascades (Fig. 11). These findings indicate that the piRNA-14633/METTL14/CYP1B1 axis plays a crucial role in promoting CC progression and may be regarded as a novel prognostic marker and possible therapeutic target of CC.

## Conclusion

The importance of piRNA in CC has rarely been tapped. In the present study, piRNA-14633 was found to be significantly upregulated in CCT and CC cells, and we also demonstrated that piRNA-14633 contributed to proliferation, migration and invasion by increasing METTL14 expression and m6A RNA methylation. Further studies should follow to understand the deeper mechanism of piRNA-14633 regulating m6A RNA methylation in CC. In short, pi-14633 plays a crucial role in promoting malignancy in CC via the METTL14/CYP1B1 pathway, suggesting that targeting the piRNA-14633/METTL14/CYP1B1 axis with selective inhibitors could represent a promising therapeutic strategy for treating CC.

## Supplementary Information

**Additional file 1: Table S1.** Primer sequences for RT-qPCR.

**Additional file 2: Fig. S1.** Effect of piRNA-14633 mimic/inhibitor on piRNA-14633 expression. **a** Real-time PCR of piRNA-14633 expression in Caski cells treated with piRNA-14633 mimic or inhibitor. **b** Real-time PCR of piRNA-14633 expression in Siha cells treated with 50 nM piRNA-14633 mimic or inhibitor. *P < *0.05* vs. mi-NC group. ^#^*P* < *0.05* vs. in-NC group. mi-14633 piRNA-14633 mimic, in-14633 piRNA-14633 inhibitor.

## Data Availability

The datasets generated/analyzed during the current study are available.
