# Supplementary material for: piRNA-14633 promotes cervical cancer cell malignancy in a METTL14-dependent m6A RNA methylation manner
Source: J Transl Med. 2022 Jan 29;20:51. doi: 10.1186/s12967-022-03257-2 (PMC8802215; doi:10.1186/s12967-022-03257-2)
Supplement: Supplementary file 1 — Additional file 1: Table S1. Primer sequences for RT-qPCR. [file 12967_2022_3257_MOESM1_ESM.doc]

**Table. S1 Primer sequences for RT-qPCR**

| **Target** | **Primer sequences（5' to 3'）** |
| --- | --- |
| **ALKBH5 Forward primer** | **TTTTCCCCCTTTAGTCTCC** |
| **ALKBH5 Reverse primer** | **CCCTTCACCAACTCCCAT** |
| **WTAP Forward primer** | **AGATGACCAACGAAGAAC** |
| **WTAP Reverse primer** | **CTAGTCGCATTACAAGGAT** |
| **FTO Forward primer** | **GGTGTCCCAAGAAATCGT** |
| **FTO Reverse primer** | **CTGGTGGCAGGAAAGAGT** |
| **GAPDH Forward primer** | **AAGGTGAAGGTCGGAGTCAA** |
| **GAPDH Reverse primer** | **GGAAGATGGTGATGGGATTT** |
| **METTL3 Forward primer** | **GAGTGTCGGAGGTGATT** |
| **METTL3 Reverse primer** | **AGTACGGGTATGTTGAGC** |
| **METTL14 Forward primer** | **TGTACTTACAAGCCGATAT** |
| **METTL14 Reverse primer** | **TAGCAGTGATGCCAGTT** |
| **CYP1B1 Forward primer** | **TGCCTGTCACTATTCCTCATGCCA** |
| **CYP1B1 Reverse primer** | **TCTGCTGGTCAGGTCCTTGTTGAT** |
