# Supplementary figures and images for: piRNA-14633 promotes cervical cancer cell malignancy in a METTL14-dependent m6A RNA methylation manner
Source: J Transl Med. 2022 Jan 29;20:51. doi: 10.1186/s12967-022-03257-2 (PMC8802215; doi:10.1186/s12967-022-03257-2)

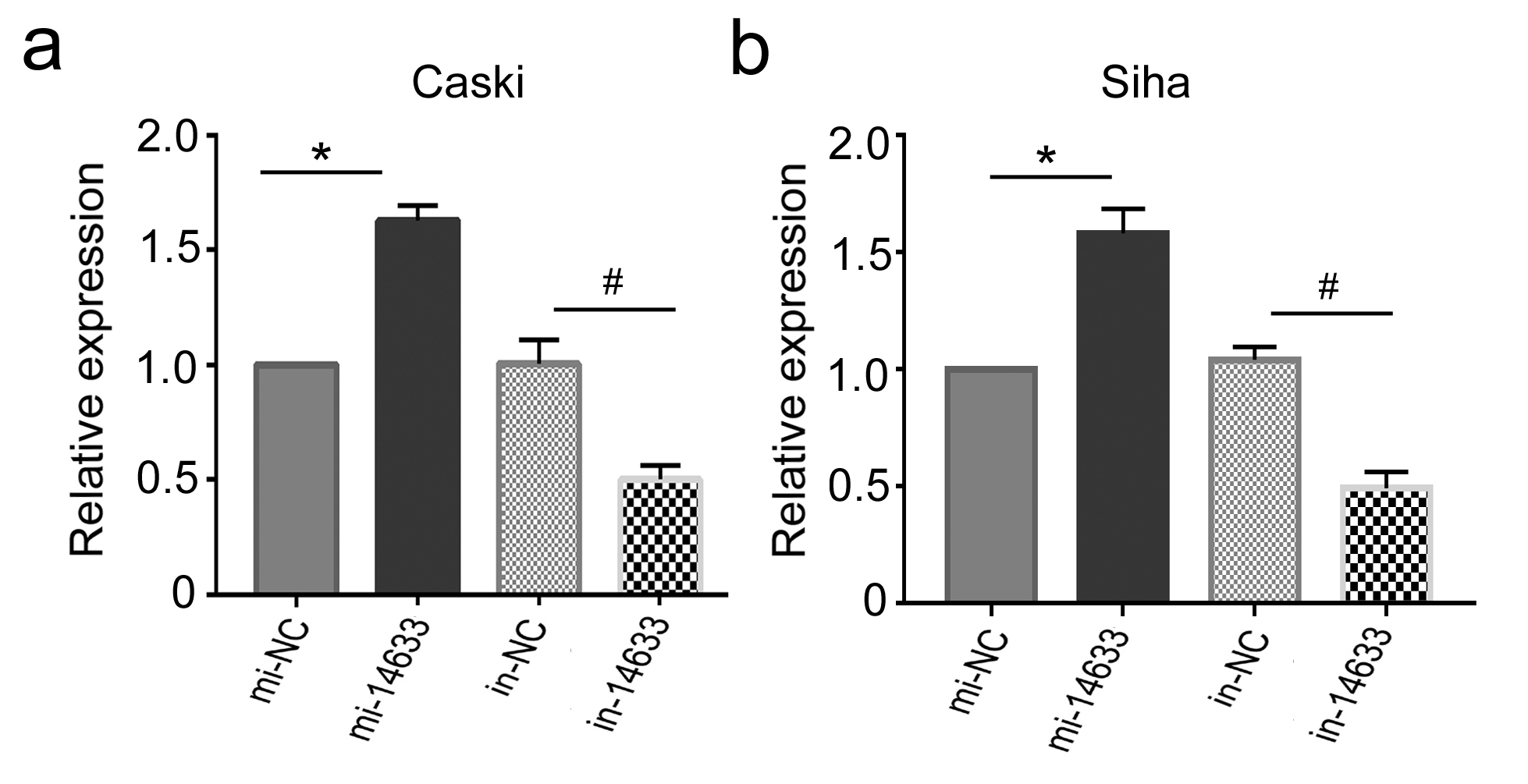

Supplement: Supplementary file 2 — Additional file 2: Fig. S1. Effect of piRNA-14633 mimic/inhibitor on piRNA-14633 expression. a Real-time PCR of piRNA-14633 expression in Caski cells treated with piRNA-14633 mimic or inhibitor. b Real-time PCR of piRNA-14633 expression in Siha cells treated with 50 nM piRNA-14633 mimic or inhibitor. *P < 0.05 vs. mi-NC group. #P < 0.05 vs. in-NC group. mi-14633 piRNA-14633 mimic, in-14633 piRNA-14633 inhibitor. [file 12967_2022_3257_MOESM2_ESM.jpg]
